# Proteasomal-Mediated Degradation of AKAP150 Accompanies AMPAR Endocytosis during cLTD

**DOI:** 10.1523/ENEURO.0218-19.2020

**Published:** 2020-03-31

**Authors:** Wenwen Cheng, Dolores Siedlecki-Wullich, Judit Català-Solsona, Cristina Fábregas, Rut Fadó, Núria Casals, Montse Solé, Mercedes Unzeta, Carlos A. Saura, José Rodríguez-Alvarez, Alfredo J. Miñano-Molina

**Affiliations:** 1 Institut de Neurociències and Dpt. Bioquímica i Biologia Molecular, Universitat Autònoma de Barcelona, Cerdanyola del Vallès 08193, Spain; 2 Centro de Investigación Biomédica en Red sobre Enfermedades Neurodegenerativas (CIBERNED), Madrid 28031, Spain; 3 Basic Sciences Department, Facultat de Medicina i Ciències de la Salut, Universitat Internacional de Catalunya (UIC), Sant Cugat del Vallès 08195, Spain; 4 Centro de Investigación Biomédica en Red de Fisiopatología de la Obesidad y la Nutrición (CIBEROBN), Instituto de Salud Carlos III, Madrid E-28029, Spain; 5 Neurovascular Research Laboratory, Vall d’Hebron Institute of Research, Universitat Autònoma de Barcelona, Barcelona 08035, Spain; 6 Dominick P. Purpura Department of Neuroscience, Albert Einstein College of Medicine, New York, NY 10461

**Keywords:** AKAP150, AMPAR, LTD, LTP, plasticity, trafficking

## Abstract

The number and function of synaptic AMPA receptors (AMPARs) tightly regulates excitatory synaptic transmission. Current evidence suggests that AMPARs are inserted into the postsynaptic membrane during long-term potentiation (LTP) and are removed from the membrane during long-term depression (LTD). Dephosphorylation of GluA1 at Ser-845 and enhanced endocytosis are critical events in the modulation of LTD. Moreover, changes in scaffold proteins from the postsynaptic density (PSD) could be also related to AMPAR regulation in LTD. In the present study we analyzed the effect of chemical LTD (cLTD) on A-kinase anchoring protein (AKAP)150 and AMPARs levels in mouse-cultured neurons. We show that cLTD induces AKAP150 protein degradation via proteasome, coinciding with GluA1 dephosphorylation at Ser-845 and endocytosis of GluA1-containing AMPARs. Pharmacological inhibition of proteasome activity, but not phosphatase calcineurin (CaN), reverted cLTD-induced AKAP150 protein degradation. Importantly, AKAP150 silencing induced dephosphorylation of GluA1 Ser-845 and GluA1-AMPARs endocytosis while AKAP150 overexpression blocked cLTD-mediated GluA1-AMPARs endocytosis. Our results provide direct evidence that cLTD-induced AKAP150 degradation by the proteasome contributes to synaptic AMPARs endocytosis.

## Significance Statement

Multiple evidences have shown that changes in glutamatergic synaptic AMPA receptors (AMPARs) modulate excitatory synaptic plasticity, playing a fundamental role in learning and memory-related processes such as long-term potentiation (LTP) or long-term depression (LTD). AMPARs are inserted in the synapses during LTP and removed during LTD although the mechanisms involved in synaptic AMPARs dynamics are not fully understood. Here, we show that chemical LTD (cLTD)-dependent proteasome degradation of A-kinase anchoring protein (AKAP)150, a scaffold protein from the postsynaptic density (PSD), is related to synaptic AMPARs removal during cLTD whereas restored AKAP150 levels blocks cLTD-mediated endocytosis of AMPARs. Thus, our findings expand our understanding of the regulatory role of AKAP150 on AMPARs dynamics at glutamatergic synapses.

## Introduction

NMDA receptor-dependent long-term potentiation (LTP) and long-term depression (LTD) in excitatory hippocampal synapses are believed to play a fundamental role in learning and memory. Several reports have shown that changes in synaptic AMPA receptor (AMPAR) number, properties or subunits composition are involved in LTP and LTD (Huganir and Nicoll, 2013). It is now well established that LTP involves the modulation of AMPARs that are already present at the synapse and/or the rapid recruitment of new AMPARs from extrasynaptic sites to the synapse (Benke et al., 1998; Park et al., 2004; Makino and Malinow, 2009; Petrini et al., 2009). On the other hand, removal of synaptic AMPARs from synapses and even their degradation, seem to be fundamental issues for LTD (Beattie et al., 2000; Ehlers 2000; Lee et al., 2000).

It has been described that membrane insertion of GluA1-containing AMPARs is regulated by cAMP-dependent protein kinase (PKA), Ca^2+^/calmodulin-dependent protein kinase II (CaMKII), and protein kinase C (PKC) phosphorylation of Ser-845, Ser-831, and Ser-818, respectively (Mammen et al., 1997; Song and Huganir, 2002; Esteban et al., 2003; Boehm et al., 2006). Among them, PKA-mediated phosphorylation of Ser-845 seems to be essential for new AMPARs recruitment to extrasynaptic sites and was proposed to represent a necessary event for the establishment of LTP (Esteban et al., 2003; Makino and Malinow, 2009; Lee et al., 2010). Activity-dependent AMPAR exocytosis at extrasynaptic sites during LTP also depends on the binding of complexin to a postsynaptic unique SNARE complex (Ahmad et al., 2012; Jurado et al., 2013). However, extrasynaptic AMPARs delivery and lateral diffusion to the synapse are necessary but not sufficient for LTP because AMPARs reaching the synapse are not necessarily retained (Ehlers et al., 2007; Petrini et al., 2009). Synaptic retention of AMPARs occurs mainly through binding with transmembrane AMPAR regulatory proteins (TARPs) and PDZ-containing scaffolding proteins present in the postsynaptic density (PSD) such as Stargazin or PSD95 (Ehrlich and Malinow, 2004; Kim and Sheng, 2004; Elias et al., 2006; Opazo et al., 2010).

In contrast to LTP, removal of synaptic AMPARs during LTD seems to be mediated by clathrin-dependent endocytosis (Beattie et al., 2000; Man et al., 2000) followed by activation of degradation or recycling pathways (Ehlers 2000). LTD triggered by NMDA receptor activation is Ca^2+^-dependent and requires the activation of phosphatases that dephosphorylate AMPARs and their associated proteins to promote the destabilization of synaptic AMPARs, lateral diffusion to extrasynaptic sites and endocytosis (Mulkey et al., 1994; Lee et al., 1998, 2000; Tomita et al., 2004). For instance, dephosphorylation of GluA1 Ser-845 by PP2B (calcineurin; CaN) is necessary for LTD-mediated synaptic AMPARs removal (Oh et al., 2006; Lee et al., 2010). Since PKA-mediated phosphorylation of Ser-845 is needed for LTP priming, fine-tuning of PKA/CaN activity is determinant for the insertion or removal of synaptic AMPARs during LTP/LTD. On the other hand, CaMKII is also emerging as an additional requirement for LTD, reducing synaptic location of GluA1-containing AMPAR and thus, producing synaptic depression (Lu et al., 2010; Coultrap et al., 2014).

A-kinase anchoring protein 79 (AKAP79; in human) or AKAP150 (the rodent homolog) is a postsynaptic scaffold protein that anchors PKA, PKC, and CaN to the PSD, regulating AMPAR phosphorylation, trafficking and activity associated with LTP and LTD (Tavalin et al., 2002; Smith et al., 2006; Lu et al., 2007, 2008; Tavalin, 2008; Jurado et al., 2010; Sanderson et al., 2012). AKAP79/150 is linked to both AMPAR and NMDAR by additional binding to the membrane-associated guanylate kinase (MAGUK) scaffold proteins SAP97 and PSD95 (Colledge et al., 2000; Bhattacharyya et al., 2009; Robertson et al., 2009). All of them are S-palmitoylated in an activity-dependent manner controlling their targeting to dendritic spines and regulating trafficking, spine enlargement and synaptic function (Fukata and Fukata, 2010; Keith et al., 2012; Purkey et al., 2018). Recently, it has been reported that LTD induces removal of synaptic AKAP79/150 via CaMKII (Woolfrey et al., 2018) contributing, together with the regulation of PKA/CaN, to synaptic GluA1-AMPARs removal. Thus, antagonistic regulation of PKA, CaN and CaMKII activities together with the cycling of AKAP79/150 in and out the synapses are major molecular mechanisms controlling synaptic AMPARs. However, it is still unknown whether degradation of AKAP79/150 is triggered by LTD and whether it could be associated to AMPARs endocytosis. In the present study, we show that AKAP150 is targeted to the ubiquitin-proteasome system (UPS) and degraded under chemical LTD (cLTD) conditions. Interestingly, pharmacological inhibition of proteasome restores basal AKAP150 levels and blocks AKAP150 removal from synapses. This degradation of AKAP150 goes in parallel with LTD-mediated GluA1 dephosphorylation at Ser-845 and GluA1-AMPARs endocytosis. Indeed, maintaining AKAP150 levels blocks LTD-mediated GluA1-AMPARs endocytosis.

## Materials and Methods

### Cell culture, transfection, and transduction

Dissociated hippocampal cultures were prepared from newborn C57BL/6J wild-type mice as previously described (Ahmad et al., 2012). Briefly, hippocampi were isolated and incubated with a digestion solution containing papain (10 U/ml; Worthington) and DNase I (300–450 Kunitz/ml; Sigma) for 30 min at 37°C. A papain inactivation solution containing fetal bovine serum and bovine serum albumin was then applied. Tissue was triturated and cells were plated on poly-D-lysine (0.01 mg/ml in 0.1 M borate buffer, pH 8.4; both from Sigma)-coated 24-well plates with 12-mm coverslip (Paul Marienfeld) at a density of 75,000 cells per cover slip (for imaging studies) or from 300,000 to 900,000 cells per 35- to 60-mm dishes, respectively (for Western blotting). Wells contained Neurobasal-A media (Thermo Fisher Scientific) supplemented with B-27 (Thermo Fisher Scientific) and GlutaMAX (Thermo Fisher Scientific). Glial growth was inhibited by 5-fluorodeoxyuridine (FDUR) at 3 days in vitro (DIV). Neurons were maintained in a humidified incubator at 37°C with 5% CO_2_, and half medium was replaced with fresh medium every week until use at 17–21 DIV. All animal procedures were performed in accordance with Universitat Autònoma de Barcelona animal care committee’s regulations. When necessary, neurons were transduced with lentivirus [2–3 transducing units (TU)] on day 7 in culture and assayed as indicated in results. Direct counting of GFP-positive cells was used to monitor the efficiency of infection in each experiment. The percentage of infected cells reached at least 75%. Overexpression or down-regulation of AKAP was assessed by immunoblotting and immunocytochemistry.

HEK293T cells were maintained in DMEM supplemented with 10% fetal bovine serum and 1× pen/strep (Thermo Fisher Scientific). Cells at 90% confluence were passaged every 6 d using 1× Trypsin-EDTA solution (T3924-Sigma) replacing the media every 3 d. HEK293T cells were transfected using the calcium phosphate method with 20 μg plasmid DNA.

### Plasmids and lentivirus production

AKAP150 was cloned by PCR from pcDNA3-AKAP150 vector (kindly provided by J. D. Scott; Howard Hughes Medical Institute, University of Washington, Seattle) and inserted into the lentiviral vector pWPI-IRES-GFP at the PmeI site for lentiviral overexpression experiments. pWPI vector was a gift from Didier Trono (Addgene plasmid #12 254). For RNAi interference experiments, a shAKAP150 construct was generated using the Thermo Fisher Scientific RNAi designer webtool (https://rnaidesigner.thermofisher.com/rnaiexpress/) using specific oligonucleotides against mouse AKAP150 (NM_001101471.1) indicated by capital letter as follows: RNAi, forward, 5’-agatctcccGGAAAGTGCTTTCATTCAAAGttcaagagaCTTTGAATGAAAGCACTTTCC ttttta-3′ and reverse, 5’-aagcttaaaaaGGAAAGTGCTTTCATTCAAAGtctcttgaaTTTGAATGAAAGCACTTTCCgggg-3′. Oligonulceotides were purchased at Thermo Fisher Scientific and cloned between BglII and HindIII sites of the pSUPER.retro.puro vector (OligoEngine), under the control of the DNA Pol III promoter of H1 (Brummelkamp et al., 2002). Lentiviral constructs were achieved by digestion at the EcoRI-ClaI sites to replace H1 promoter in the lentiviral vector pLVTHM with H1-short hairpin RNA cassette from pSUPER. pLVTHM-H1-RNAi was used for lentiviral knock-down experiments. pLVTHM vector was a gift from Didier Trono (Addgene plasmid #12247). Lentiviruses were produced in HEK293T cells using calcium phosphate transfection of a 20 μg of pLVTHM-shAKAP150 or 20 μg of pWPI-AKAP150 (for AKAP down-regulation or over-expression, respectively), together with 15 μg of psPax2 and 6 μg of pMD2.G (gifts from Didier Trono, Addgene, #12260 and #12259, respectively); 24, 36, and 48 h after transfection, lentivirus-containing media were harvested, filtered with 0.45 μm filter units and centrifuged at 25,000 rpm for 2 h at 4°C. Pelleted viruses were resuspended in ice-cold PBS buffer by shaking at 175 rpm overnight at 4°C. Lentiviruses were aliquoted and stored at −80°C until use. Biological titers of the viral preparations expressed as a number of TUs per milliliter were determined by infecting HEK293T cells with serial dilutions. After 48 h, the percentage of GFP-positive cells was detected by flow cytometry (Cytomics FC 500, Beckman Coulter) and analyzed with RXT software.

### Cell stimulation and lysate preparation

cLTD was induced as previously described (Oh et al., 2006). Cultures were first incubated in ACSF for 30 min at room temperature: 125 mM NaCl, 2.5 mM KCl, 1 mM MgCl_2_, 2 mM CaCl_2_, 33 mM D-glucose, and 25 mM HEPES (pH 7.3), followed by stimulation with 50 μM NMDA in ACSF (no MgCl_2_) for 5 min. After NMDA treatment, neurons were replaced in regular ACSF and then subjected to the corresponding procedure at indicated time points. Cultures were then washed with ice-cold PBS once and lysed in cold 1% NP-40 homogenization buffer (20 mM Tris, pH 7.5, 150 mM NaCl, 5 mM EDTA, 1 mM PMSF, 1 mM Na_2_VO_4_, and 1× Sigma protease inhibitor and phosphatase inhibitor cocktails). When indicated, cultures were pre-treated or post-treated with BAPTA-AM (20 μM), FK506 (10 μM), MG132 (10 μM), or MK801 (10 μM; all from Tocris) and were present until cells were lysed. cLTP was induced as previously described (Otmakhov et al., 2004) by application of forskolin (50 μM) and rolipram (0.1 μM; both from Tocris), 60 min before or after cLTD. Cells were lysed 60 min after cLTP or cLTD treatment. Lysates were centrifuged at 12,000 × *g* for 10 min at 4°C and protein in the supernatant was quantified by Bradford method assay kit (Bio-Rad Laboratories). The intensity of the cLTD protocol may vary depending on the cultures.

### Surface biotinylation

Treated cells were transferred to ice-cold PBS-Ca^2+^-Mg^2+^ buffer (pH 7.4; 1 mM CaCl_2_; 0.1 mM MgCl_2_), followed by biotinylation in 1 mg/ml of biotin (EZ-Link Sulfo-NHS-SS-Biotin; Thermo Fisher Scientific) for 30 min with slow agitation. Free biotin was quenched by wash (3×) in cold PBS-Ca^2+^-Mg^2+^ + glycine 0.1 M. Cell cultures were immediately lysed in cold 1% Triton X-100 homogenization buffer (150 μl/35 mm well; two well per condition; 50 mM NaCl, 10 mM EDTA, 10 mM EGTA, 1 mM Na_3_VO_4_, 50 mM NaF, 25 mM NaPPi, 1 mM β-glycerphosphate, 1 mM phenylmethylsulfonyl fluoride, 1× protease inhibitor cocktail, 1× phosphatase inhibitor cocktail, and 50 mM HEPES; pH 7.5). Cell lysates were centrifuged at 10,000 × *g* for 20 min to pellet insoluble fraction. A total of 75 μl of the supernatant were mixed and heated with 25 μl of 4× SDS sample buffer to determine total fraction of GluA1 (surface plus internal). Biotinylated surface proteins in the remaining supernatant (225 μl) were pulled-down with 40 μl of 50% avidin-agarose beads (ImmunoPure Immobilized Avidin; Thermo Fisher Scientific) overnight at 4°C. The beads were pelleted and 75 μl of the supernatant (internal fraction) were mixed and heated with 25 μl of 4× SDS sample buffer. The beads were then rinsed three times with 1% Triton X-100 homogenization buffer and heated in 100 μl of 2× SDS sample buffer (surface fraction). Equal volumes of total and biotinylated fractions were subjected to 10% SDS-PAGE, probed by immunoblot for total GluA1 levels and normalized to GAPDH.

### Immunoprecipitation

Neurons were washed in ice-cold PBS 1× and immediately lysed in cold immunoprecipitation buffer (200 μl × 60 mm plate; four plates per condition; 0.1% SDS, 1% Triton X-100, 1 mM EDTA, 1 mM EGTA, 50 mM NaF, 5 mM NaPPi, 10 mM N-ethylmaleimide, 1 × protease inhibitor cocktail, and 1 × phosphatase inhibitor cocktail). Homogenates from cultures were centrifuged at 16,000 × *g* for 10 min at 4°C and the protein in the supernatant was quantified by BCA protein assay kit (Bio-Rad Laboratories); 50 μg of protein from each supernatant were mixed and heated with 4× SDS sample buffer to keep total homogenate fraction (1 μg/μl). The remaining supernatants were immunoprecipitated with 5 μg of either rabbit anti-AKAP150 (clone R-300; sc-10 765; RRID: AB_2289482, Santa Cruz Biotechnology) or control rabbit IgG (011-000-003; RRID: AB_2337118; Jackson ImmunoResearch) antibodies overnight at 4°C, with gentle rocking. Immune complexes were precipitated for 1 h at 4°C using 40 μl protein G Sepharose beads (GE Healthcare Life Sciences). The beads were pelleted, washed and denatured in 100 μl of 2× SDS sample buffer, and 20 μl were loaded on an SDS-PAGE gel for immunoblotting.

### Immunoblotting

Samples were separated on 7.5% or 10% SDS-PAGE and transferred onto Hybond-C Extra, Nitrocellulose membranes (GE Healthcare Life Sciences). Blots were blocked at room temperature for 1 h with 10% dry milk, 0.1% BSA (fraction V), pH 7.4 in PBS and incubated at 4°C overnight with primary antibody in PBS 0.1% BSA, pH 7.4. Primary antibodies were: anti-phospho-Ser-845-GluA1 (1:1000; clone EPR2148; 04–1073; RRID: AB_1977219; Merck-Millipore), anti-GluA1 (1:1000; AB1504; RRID: AB_2113602; Merck-Millipore), anti-AKAP150 (1:1000; 07–210; RRID: AB_310430; Merck-Millipore), anti-PSD95 (1:1000; clone 6G6-1C9; ab2723; RRID: AB_303248; Abcam), anti-SAP97 (1:1000; clone K64/15; 75–030; RRID: AB_2091920; UC Davis/NIH NeuroMab), anti-Ubiquitin (1:5000; U5379; RRID: AB_477667), anti-β-tubulin (1:20,000; clone 5H1; 556321; RRID: AB_396360; BD Biosciences), anti-β-actin (1:20,000; clone AC-74; A2228; RRID: AB_476697; Sigma), and anti-GAPDH (1:40 000; clone 6C5; AM4300; RRID: AB_437392; Thermo Fisher Scientific). After washing, blots were incubated with horseradish peroxidase-conjugated secondary antibodies, goat anti-mouse or goat anti-rabbit (554002 and 554021; RRID: AB_395198 and RRID: 395213, respectively; BD Biosciences), diluted in blocking buffer, and developed using the ECL Western Blotting Detection Reagents (GE Healthcare Life Sciences). Semi-quantitative analysis of immunoblots was performed by densitometry using ImageJ 2.0v (https://imageJ.nih.gov/ij/) and protein levels were corrected for corresponding loading control.

### Immunocytochemistry

Neuronal cultures on coverslips were washed with ice-cold 1× PBS, fixed with ice-cold 4% paraformaldehyde with 4% sucrose in 1× PBS for 15 min at 4°C and then permeabilized with 0.1% Triton X-100 for 20 min at room temperature. For surface GluA1-containing AMPARs detection, no permeabilization was done. Afterwards, plates were washed twice with PBS, blocked with 2% normal Goat serum (Sigma) in 1× PBS (blocking buffer) for 1 h at 37°C and then incubated for 1 h at 37°C with specific antibodies detecting AKAP150 (1:200; clone N-19; sc-6446; RRID: AB_2225903 and 1:200; clone R-300; sc-10 765; AB_2289482, Santa Cruz Biotechnology), PSD95 (1:1000; clone 6G6-1C9; ab2723; RRID: AB_303248; Abcam), GluA1 (1:1000; AB1504; RRID: AB_2113602; Merck-Millipore), GFP (1:1000; ab13970; RRID: 300798; Abcam), and VGLUT1 (1:500; AB5905; RRID: 2301751; Merck-Millipore). Surface GluA1-containing AMPARs were measured using a rabbit polyclonal antibody directed against the N terminus of the GluA1 subunit (1:30; PC246; RRID: 564636; Merck-Millipore). Neurons were washed with warmed PBS-MC-sucrose (PBS 1×, 0.5 mM CaCl_2_, 1 mM MgCl_2_, 4% sucrose) and incubated with N-term-GluA1 antibody in PBS-MC-sucrose at 37°C for 5 min. Cells were then washed with ice-cold PBS-MC and fixed with ice-cold 4% paraformaldehyde with 4% sucrose in 1× PBS for 15 min at 4°C. Next, plates were washed twice with ice-cold PBS-MC, blocked for 1 h, and incubated with Alexa Flour-conjugated secondary antibody (1:300) in blocking buffer for 1 h, both at room temperature. Cells were washed, postfixed with methanol at −20°C for 1 min, blocked, and incubated with specific antibodies against desired protein. Overall, primary antibodies were detected after washing with 1× PBS by incubation (1 h at 37°C) with Alexa Fluor-conjugated secondary antibodies (1:500 or 1:1000; A405, A488, A568 and/or A633; Thermo Fisher Scientific) in blocking buffer. Cells were washed in 1× PBS and when necessary, treated with HOECHST 33258 for nuclei staining (1:10,000 in PBS; H3569; Thermo Fisher Scientific). Following, coverslips were washed with 1× PBS, and mounted on glass slides on Fluoromount-G as an anti-quenching reagent (Southern Biotech).

### Confocal imaging and analysis

Confocal z-stacks were acquired on a Zeiss LSM700 confocal microscope using the 63×/1.40 NA oil objective or Olympus FV3000 confocal microscope using the 60×/1.5 NA oil High Resolution objective. Sequential frame acquisition was set to acquire an average of 10 planes per stack at 16 bits and a minimum of 1024 × 1024 resolution. Channel gain settings were optically adjusted to minimize saturation of punctae and were maintained across experimental groups. Unmodified images were used for all analyses that were done blind and linear scaling was applied on images only for presentation purposes using ImageJ software. Fluorescent signal on the single planes from a stack, quantification of punctae number and punctae integrated fluorescence intensity over area measurements were performed with ImageJ software. Untreated and treated cells from the same culture preparation were always compared. For each experimental group an average of > 200 μm dendrite was quantified for 8–12 images per experiment in duplicate and repeated for a total of three or four independent experiments.

### Statistical analysis

Statistical analysis was performed with Prism 6.0 (GraphPad Software). One-way ANOVA with Bonferroni’s multiple comparison *post hoc* test or unpaired Student’s *t* test was used for mean comparisons between experimental conditions as required. Differences were considered significant when *p* < 0.05. Depending on the type of experiment the number of samples used for statistical analyses, *n* refers to the accumulated number of dendrites, punctae, or independent cultures assayed per group (as indicated in figure legend) being always three or more independent biological replicates. Data in bar graphs are reported as mean ± SEM.

## Results

### cLTD triggers a decrease in Ser-845 GluA1 phosphorylation, GluA1 protein levels, GluA1-AMPAR surface expression, and AKAP150 degradation

**Figure 1.**
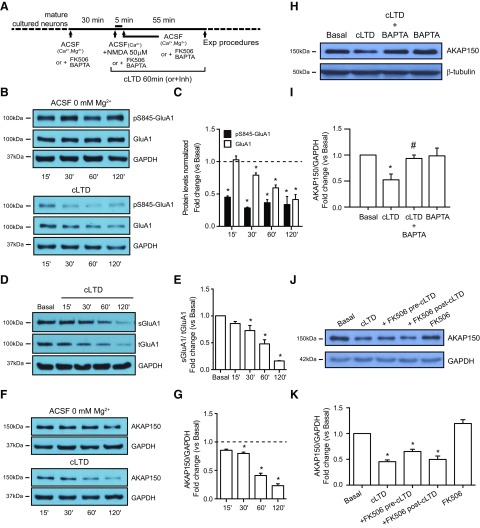
cLTD reduces AKAP150. ***A***, Cultured neurons were incubated in ACSF buffer for 30 min to equilibrate cells before 50 μM NMDA treatment for 5 min (cLTD). Cells were lysed after cLTD induction at indicated times (or 60 min if no time is indicated) in the presence or absence of inhibitors, antagonists, or cell-permeable Ca^2+^ chelators as indicated in the experimental design scheme. ***B***, Representative blot images showing reduced levels of Ser-845 GluA1 and GluA1 after treatment (cLTD) compared with cells in ACSF buffer. Ser-845 GluA1 levels (pS845-GluA1; ∼110-kDa band, top panel) were related to total GluA1 (GluA1; ∼110-kDa band, middle panel). GAPDH was used as a loading control (∼36-kDa band, bottom panel). ***C***, Quantification of pS845-GluA1 and GluA1 changes in response to NMDA stimulation compared with basal condition (*n* = 5–17, *p <* *0.0001 vs basal). ***D***, Representative blot images showing reduced levels of surface GluA1 after treatment (sGluA1; ∼110-kDa band, top panel) related to total GluA1 (tGluA1; ∼110-kDa band, middle panel). GAPDH was used as a loading control (∼36-kDa band, bottom panel). ***E***, Quantification of surface GluA1 changes in response to cLTD compared with basal condition (*n* = 6, *p =* *0.0310 for 30 min and *p <* *0.0001 for 60 min and 120 min vs basal). ***F***, Representative blot images showing reduced levels of AKAP150 after cLTD (∼150-kDa band, top panel) related to GAPDH (bottom panel). ***G***, Quantification of AKAP150 changes in response to cLTD compared with baseline (*n* = 5–20, *p =* *0.0021 for 30 min and *p <* *0.0001 for 60 min and 120 min vs basal). ***H***, ***J***, Representative blot images showing the effect of the presence of Ca^2+^ chelator BAPTA (***H***; 20 μM) or Ca^2+^-dependent phosphatase CaN inhibitor FK506 (***J***; 10 μM) on AKAP150 protein levels after cLTD (top panel) related to β-tubulin (∼51-kDa band, bottom panel) or GAPDH (bottom panel) used as a loading control. ***I***, ***K***, Quantification of AKAP150 changes in response to cLTD in presence of BAPTA (***I***) or FK506 (***K***) compared with cLTD treatment (*n* = 3 in BAPTA experiments, *p =* *0.0187 vs basal and #*p *=* *0.0377 vs cLTD, *n* = 3–10 in FK506 pre-cLTD, *p <* *0.0001 vs basal and FK506 post-cLTD experiments, *p =* *0.0073 vs basal). Bar represent mean ± SEM.

First, we analyzed different cLTD paradigms used in the literature, changing NMDA dose and time of NMDAR stimulation, to observe the characteristics features of Ser-845-GluA1 dephosphorylation and decrease of GluA1-AMPARs levels (Kamal et al., 1999; Oh et al., 2006; Bhattacharyya et al., 2009; Li et al., 2010; Keith et al., 2012; Shehata et al., 2012; Bacaj et al., 2015; Yap et al., 2017). Since consistent results (data not shown) were observed when hippocampal cultures were treated with 50 μM NMDA for 5 min in 0 mM Mg^2^, we decided to use these conditions in our experiments. Western blot analysis revealed a time-dependent decrease of Ser-845 GluA1 phosphorylation in hippocampal cultured neurons after cLTD induction [50 μM for 5 min; cLTD (Fig. 1*A–C*); cLTD: 0.449 ± 0.020 for 15 min, 0.283 ± 0.014 for 30 min, 0.365 ± 0.047 for 60 min, and 0.335 ± 0.125 for 120 min; *p <* *0.0001, respectively, vs basal) in accordance to previously reported data (Beattie et al., 2000; Ehlers 2000; He et al., 2011). Moreover, significant time-dependent changes were also found in GluA1 total levels after NMDA stimulus (cLTD: 0.791 ± 0.034 for 30 min; *p =* *0.0189, 0.594 ± 0.045 for 60 min and 0.415 ± 0.077 for 120 min; *p <**0.0001, respectively, vs basal) as previously reported (Fernández-Monreal et al., 2012). A reduction in surface GluA1-containing AMPARs after NMDA application was also observed by biotinylation and Western blot analyses. The surface reduction in GluA1-containing AMPARs was also time-dependent starting with 30-min NMDA application (Fig. 1*D*,*E*; 0.726 ± 0.095 for 30 min; *p =* *0.0310, 0.478 ± 0.077 for 60 min and 0.161 ± 0.005 for 120 min; *p <* *0.0001, respectively, vs basal). Previous reports showed that cLTD causes a redistribution of the scaffolding protein AKAP150 away from synapses (Gomez et al., 2002). However, it has not been reported whether this redistribution is also associated to changes in total AKAP150 levels. Our results indicate that cLTD significantly reduces AKAP150 levels in a time-dependent manner, as shown in the immunoblot analysis (Fig. 1*F*,*G*; cLTD: 0.799 ± 0.024 for 30 min; *p =* *0.0021, 0.410 ± 0.041 for 60 min, 0.232 ± 0.034 for 120 min; *p <* *0.0001 vs basal). As expected, application of Ca^2+^ chelator BAPTA-AM blocked cLTD-mediated AKAP150 levels decrease (Fig. 1*H*,*I*; cLTD: 0.525 ± 0.111; cLTD + BAPTA: 0.935 ± 0.066; *p =* *0.0187 vs basal, #*p *=* *0.0377 vs cLTD).

Moreover, the phosphatase CaN has been described to form a complex with AKAP150 that is required for cLTD (Jurado et al., 2010) and to play a major role in AMPAR endocytosis by dephosphorylation of GluA1 Ser-845 (Oh et al., 2006; Lee et al., 2010). Therefore, we examined whether CaN activity is necessary for cLTD NMDA-dependent AKAP150 degradation. Pharmacological inhibition of CaN with FK506 after or during cLTD induction did not prevent the degradation of AKAP150 (Fig. 1*J*,*K*; cLTD: 0.454 ± 0.034; cLTD + FK506 post-cLTD: 0.652 ± 0.043; *p=* *0.0073 vs basal; cLTD + FK506 pre-cLTD: 0.499 ± 0.066; *p <* *0.0001 vs basal). These results confirm that although CaN mediates cLTD-dependent AKAP150 redistribution away from the synapse (Smith et al., 2006), it is not involved in cLTD-dependent AKAP150 degradation.

### Ubiquitin-proteasome system mediates cLTD-dependent AKAP150 degradation

**Figure 2.**
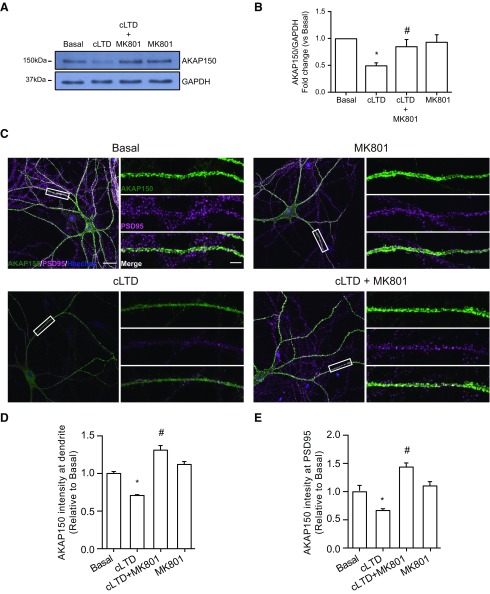
cLTD-mediated reduction of AKAP150 requires calcium influx through NMDA receptors. ***A***, Representative blot images showing the effect of NMDAR antagonist MK801 (10 μM) on AKAP150 protein levels after cLTD (top panel) related to GAPDH (bottom panel) used as a loading control. ***B***, Quantification of AKAP150 changes in response to NMDA stimulation in presence of MK801 compared with cLTD (*n* = 4, *p =* *0.0036 vs basal and #*p *=* *0.0395 vs cLTD). ***C***, Representative confocal images of primary neurons showing AKAP150 (green), postsynaptic marker PSD95 (magenta) and nuclei (blue) staining. Separated color panels for individual marker from the boxed regions have been magnified for dendrite clarity (right panels). Lower right panels show the merge for AKAP150 (green)/PSD95 (magenta), where cLTD reduced dendritic and synaptic intensity in AKAP150 staining. The presence of MK801 (10 μM) blocks this reduction. ***D***, Quantification of AKAP150 intensity at dendrites. cLTD reduced AKAP150 dendritic intensity and the presence of MK801 avoids this reduction (*n* > 15 dendrites of different neurons from four independent cultures, *p <* *0.0001 vs basal; #*p *<* *0.0001 vs cLTD). ***E***, Quantification of AKAP150 intensity at the PSD95. cLTD reduction of synaptic AKAP150 intensity was blocked by MK801 (*n* > 50 puncta from >15 dendrites from four independent cultures, *p =* *0.0150 vs basal; #*p *<* *0.0001 vs cLTD). Scale bars = 20 μm (***C***, left panels) and 5 μm (***C***, right panels). Bars represent mean ± SEM.

**Figure 3.**
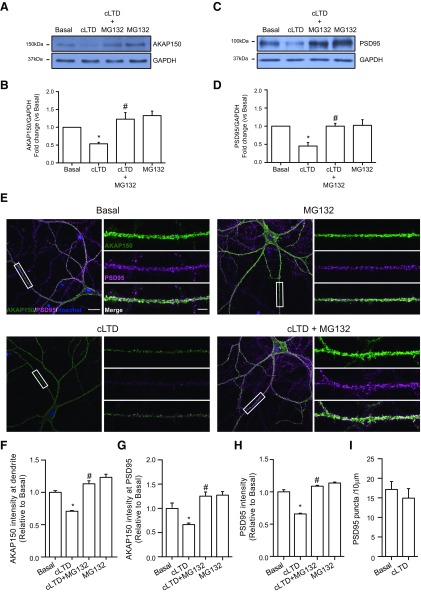
cLTD-mediated reduction of AKAP150 requires proteasome activity. ***A***, Representative blot images showing the effect of proteasome inhibitor MG132 (10 μM) on AKAP150 protein levels after cLTD (top panel) related to GAPDH (bottom panel) used as loading control. ***B***, Quantification of AKAP150 changes in response to NMDA stimulation in presence of MG132 compared with cLTD (for *n* = 4, *p =* *0.0125 vs basal and #*p *=* *0.0014 vs cLTD). ***C***, Representative blot images showing the effect of proteasome inhibitor MG132 (10 μM) on PSD95 protein levels after cLTD (top panel) related to GAPDH (bottom panel) used as loading control. ***D***, Quantification of PSD95 changes in response to NMDA stimulation in presence of MG132 compared with cLTD (for *n* = 3, *p =* *0.0092 vs basal and #*p *=* *0.0091 vs cLTD). ***E***, Representative confocal images of primary neurons showing AKAP150 (green), postsynaptic marker PSD95 (magenta) and nuclei (blue) staining. Separated color panels for individual marker from the boxed regions have been magnified for dendrite clarity (right panels). Lower right panels show the merge for AKAP150 (green)/PSD95 (magenta), where cLTD reduced dendritic and synaptic intensity in AKAP150 staining. The presence of MG132 (10 μM) blocks this reduction. ***F***, Quantification of AKAP150 intensity at dendrites. cLTD reduced AKAP150 dendritic intensity and the presence of MG132 avoids this reduction (*n* > 15 dendrites of different neurons from four independent cultures, *p <* *0.0001 vs basal; #*p *<* *0.0001 vs cLTD). ***G***, Quantification of AKAP150 intensity at the PSD95. cLTD reduction of synaptic AKAP150 intensity was blocked by MG132 (*n* > 50 puncta from >15 dendrites from four independent cultures, *p =* *0.0150 vs basal; #*p *<* *0.0001 vs cLTD). ***H***, Quantification of PSD95 intensity at dendrites. cLTD reduction of PSD95 intensity was blocked by MG132 (*n* > 15 dendrites of different neurons from four independent cultures, *p =* *0.0005 vs basal; #*p *=* *0.0002 vs cLTD). ***I***, Quantification of PSD95 puncta at dendrites normalized to basal. There is no difference in puncta number between basal and cLTD conditions (*n* > 50 puncta from >15 dendrites from four independent cultures). Scale bars = 20 μm (***A***, left panels) and 5 μm (***A***, right panels). Bars represent mean ± SEM.

**Figure 4.**
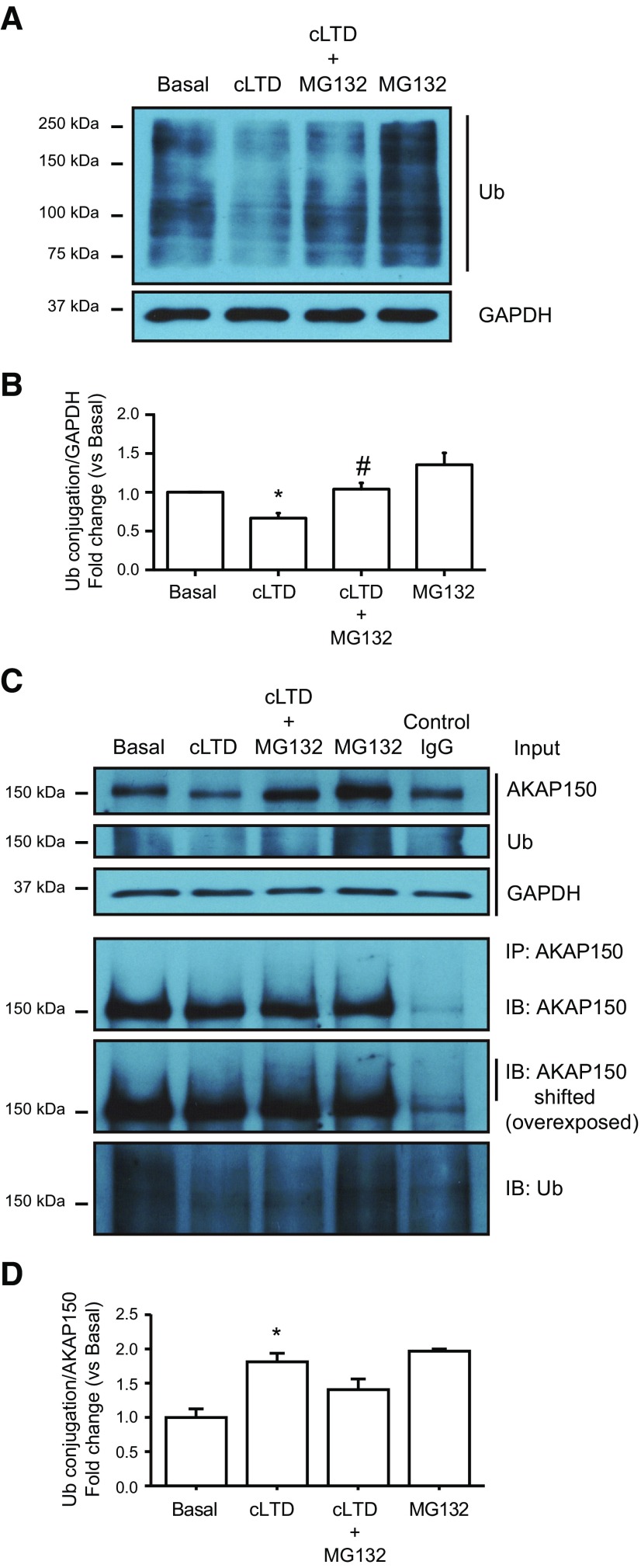
cLTD-mediated AKAP150 degradation requires AKAP150 ubiquitination. ***A***, Representative blot images showing the ubiquitination (Ub) of high molecular weight protein after cLTD (top panel) related to GAPDH (bottom panel) used as a loading control. ***B***, Quantification of total ubiquitination signal in response to cLTD in presence of MG132 (10 μM) compared with cLTD (*n* = 6, *p =* *0.0355 vs basal and #*p *=* *0.0183 vs cLTD). ***C***, Representative blot images showing an increase in AKAP150 ubiquitination after cLTD. Lysates from neurons after cLTD were immunoprecipitated with anti-AKAP150 or control rabbit IgG (control IgG) and blotted with anti-AKAP150 and anti-ubiquitin (Ub). The three top panels show representative blot images (input) for AKAP150 and 150-kDa ubiquitinated proteins (Ub) related to GAPDH (bottom panel of three) used as a loading control. The three bottom panels show representative blot images of immunoprecipitated Ub-AKAP150 protein (IP:AKAP150; including overexposed panel to observe AKAP150-shifted) and total ubiquitination levels (Ub). ***D***, Quantification of ubiquitinated-AKAP150 levels related to total AKAP150 signal in response to cLTD compared with basal (*n* = 3 for each group, *p =* *0.0026 vs basal). Bars represent mean ± SEM.

**Figure 5.**
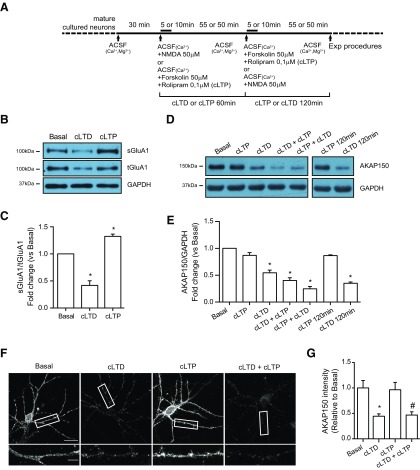
cLTP neither rescue nor prevents cLTD-mediated reduction of AKAP150 levels. ***A***, Hippocampal neurons were cultured during 17–21 DIV. cLTP was induced with forskolin/rolipram (50 μM/0.1 μM) for 60 min (10-min stimulation plus 50-min incubation in ACSF buffer) before or after cLTD as indicated in experimental design scheme. ***B***, Representative blot images showing increased levels of surface GluA1 after cLTP treatment (sGluA1; ∼110-kDa band, top panel) related to total GluA1 (tGluA1; ∼110-kDa band, middle panel). GAPDH was used as a loading control (∼36-kDa band, bottom panel). ***C***, Quantification of surface GluA1 changes in response to cLTD and cLTP compared with basal condition (*n* = 4–9, *p <* *0.0001 for cLTD and *p =* *0.0009 for cLTP vs basal). ***D***, Representative blot images showing levels of AKAP150 in response to cLTP before and after cLTD treatment (top panel) and GAPDH (bottom panel) as a loading control. ***E***, Quantification of AKAP150 changes in response to cLTP before and after cLTD treatment compared with basal (*n* = 3–9, *p <* *0.0001 vs basal). ***F***, Dendritic staining for AKAP150 after cLTD with or without cLTP. Representative confocal images showing AKAP150 (gray). Separated panels from the boxed regions have been magnified for dendrite clarity (bottom panels). cLTD reduced AKAP150 staining. The presence of cLTP (50 μM; 0.1 μM) does not rescue AKAP150 levels. ***G***, Quantification of AKAP150 intensity at dendrites normalized to basal condition. cLTD reduced AKAP150 dendritic intensity and cLTP induction does not prevent this reduction (*n* > 25 dendrites of different neurons from three independent cultures, *p =* *0.0004 vs basal; #*p *=* *0.0017 vs basal). Scale bars = 20 μm (***F***, top panels) and 5 μm (***F***, bottom panels). Bars represent mean ± SEM.

After our finding that cLTD produced a decrease in AKAP150 levels we wondered whether this effect was mediated by proteasome activation. The reduction in AKAP150 levels was blocked by pretreatment with the NMDA antagonist MK801 (Fig. 2*A*,*B*; cLTD + MK801: 0.847 ± 0.133; #*p *=* *0.0395 vs cLTD) and also by the specific inhibition of proteasome activity with MG132 (Fig. 3*A*,*B*; cLTD + MG132: 1.228 ± 0.184; #*p *=* *0.0014 vs cLTD). Since cLTD-induced NMDAR activation causes a persistent loss of synaptic AKAP150 in cultured neurons (Smith et al., 2006), we analyzed whether changes in AKAP150 levels go beyond synapse relocation and whether its degradation is participating on AKAP150 removal from synapses. We assessed AKAP150 levels by confocal microscopy pretreating neurons with both MK801 and MG132. We observed a significant reduction in AKAP150 immunoreactivity at dendrites after cLTD that was blocked by MK801 and MG132 application (Figs. 2*C*,*D*, 3*E*,*F*; cLTD: 0.504 ± 0.042; *p <* *0.0001; cLTD + MK801: 1.31 ± 0.061; #*p *<* *0.0001 vs cLTD; cLTD + MG132: 1.140 ± 0.0471; #*p *<* *0.0001 vs cLTD). Moreover, we analyzed AKAP150 levels at PSD95 clusters and we observed that both MK801 and MG132 were able to maintain AKAP150 at synapses after cLTD (Figs. 2*C*,*E*, 3*E*,*G*; cLTD: 0.666 ± 0.031; *p =* *0.0150; cLTD + MK801: 1.435 ± 0.073; #*p *<* *0.0001 vs cLTD; cLTD + MG132: 1.239 ± 0.086; #*p *<* *0.0001 vs cLTD). Previous work by Colledge et al., (2003) indicated that cLTD treatment of cultured neurons induces proteasomal degradation of PSD95, leading its decreased synaptic clustering and contributing to the removal of synaptic AKAP150 and AMPARs (Colledge et al., 2003; Smith et al., 2006). Therefore, we analyzed PSD95 levels after cLTD by Western blotting and we observed that protein levels were reduced and treatment with MG132 was able to restore them (Fig. 3*C*,*D*; cLTD + MG132: 1.001 ± 0.078; #*p *=* *0.0091 vs cLTD). We also quantified both PSD95 cluster intensity and punctae number after cLTD treatment of cultured hippocampal neurons. Consistent with previously reported data (Colledge et al., 2003; Smith et al., 2006; Bhattacharyya et al., 2009), we observed a significant decrease in PSD95 cluster intensity after 1 h of treatment (Fig. 3*E*,*H*; cLTD: 0.659 ± 0.011; *p =* *0.0005 vs basal) that was not observed in the presence of MG132 (Fig. 3*E*,*H*; cLTD + MG132: 1.088 ± 0.013; #*p *=* *0.0002 vs cLTD). However, we did not observe changes in PSD95 punctae number at dendrites after cLTD treatment (Fig. 3*I*; basal: 17.164 ± 2.002 punctae vs cLTD: 14.929 ± 2.428 punctae per 10 μm). Our data in cultured neurons suggest that not only redistribution of AKAP150 but also its degradation may play a role in the early stages of LTD. To determine whether cLTD-mediated AKAP150 decrease required AKAP150 ubiquitination, we check ubiquitination levels after cLTD induction in the presence of MG132 in cultured neurons. Surprisingly, we observed less ubiquitin-conjugated pattern after cLTD induction in whole lysate compared with basal levels (Fig. 4*A*,*B*; cLTD: 0.666 ± 0.066; *p =* *0.0355), despite we expected an increase in ubiquitination levels since inhibition of ubiquitination attenuates NMDA-dependent cLTD (Citri et al., 2009). Proteasome inhibition with MG132 restored ubiquitination levels (Fig. 4*A*,*B*; cLTD+MG132: 1.039 ± 0.079; #*p *=* *0.0183 vs cLTD), suggesting that cLTD could be inducing rapid protein degradation via ubiquitin-proteasome system. Previous studies surveying substrates of ubiquitin ligation in the PSD identified AKAP150 as a highly ubiquitinated protein under basal conditions which is highly regulated by synaptic activity (Ehlers 2003; Jia et al., 2008). Accordingly, while cLTD reduced AKAP150 levels (Fig. 4*C*; cLTD: 0.614 ± 0.059; *p =* *0.0121 vs basal), pull-down results showed a significant increase in total ubiquitin conjugated-AKAP150 observed in AKAP150 shift when overexposed (Fig. 4*C*,*D*; cLTD: 1.813 ± 0.125; *p =* *0.0026 vs basal). These results suggest that synaptic AKAP150 degradation by ubiquitin-proteasome system could be an important factor in cLTD-induced synapse regulation. It is known that AKAP150 participates in the regulation of LTP processes and even it expression is upregulated in this conditions (Génin et al., 2003; Sanderson and Dell'Acqua, 2011). Since our results show that cLTD induce AKAP150 degradation, we studied whether LTP could restore AKAP150 levels in our neuronal cultures. For this purpose we used a chemical stimulation protocol of forskolin and rolipram (Fig. 5*A*; cLTP; 50 μM /0.1 μM) that results in LTP (Grey and Burrell, 2008) and produces and increase in both GluA1 phosphorylation at Ser-845 and cell surface GluA1 levels (Fig. 5*B*,*C*; cLTD: 0.418 ± 0.085; *p <* *0.0001; cLTP 1.325 ± 0.041; *p =* *0.0009 vs basal; Oh et al., 2006; Miñano-Molina et al., 2011). When cLTP was induced in cultured neurons after cLTD no reversion of cLTD-mediated effects on AKAP150 levels was observed by immunoblotting (Fig. 5*D*,*E*; cLTD: 0.545 ± 0.049; cLTD + cLTP: 0.400 ± 0.052; *p <* *0.0001 vs basal) or by immunocytochemistry (Fig. 5*F*,*G*; cLTD: 0.444 ± 0.046; *p =* *0.0004 vs basal; cLTD + cLTP: 0.467 ± 0.065; #*p *=* *0.0017 vs basal). Similarly, AKAP150 was not observed even when we tried to favor AKAP150 retention at the synapses by inducing cLTP before cLTD (Fig. 5*D*,*E*; cLTP: 0.869 ± 0.051; cLTP + cLTD: 0.245 ± 0.044; *p <* *0.0001 vs basal).

### Endogenous AKAP150 knock-down triggers dephosphorylation and endocytosis of AMPARs

**Figure 6.**
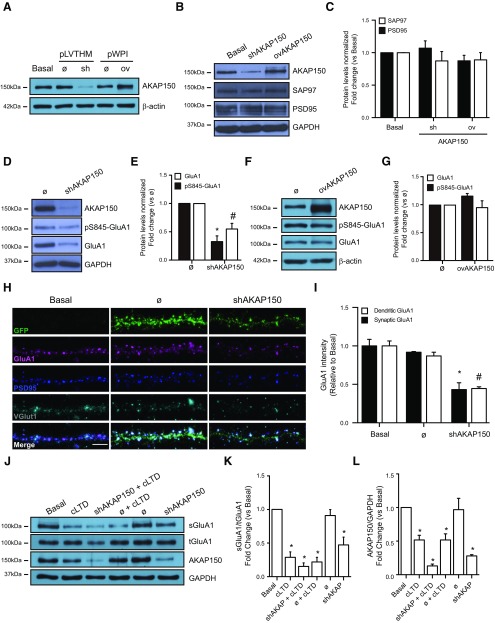
Silencing endogenous AKAP150 reduces AMPARs-GluA1 subunit levels. ***A***, AKAP150 was silenced (sh or shAKAP150) or overexpressed (ov or ovAKAP150) in neuronal cultures after transduction with lentiviral vectors. Representative blot images showing the efficiency of shAKAP150 or ovAKAP150 transduction in primary neurons. Empty lentiviral vector (Ø; pLVTHM or pWPI) was used as a control. ***B***, Silencing AKAP150 does not affect related scaffold proteins like PSD95 (∼95-kDa band) or SAP97 (∼140-kDa band). Representative blot images showing levels of AKAP150 (top panel; as a control of silencing or overexpressing), PSD95 and SAP97 when endogenous AKAP150 was knocked down (shAKAP150) or overexpressed (ovAKAP150). GAPDH (bottom panel) was used as a loading control. ***C***, Quantification of PSD95 or SAP97 levels when AKAP150 was silenced (shAKAP150) or overexpressed (overAKAP150) normalized to basal (*n* = 4). ***D***, Endogenous AKAP150 silencing reduces S845-GluA1 phosphorylation and total GluA1 protein. Representative blot images showing AKAP150 levels (top panel; as a control of silencing), phosphorylated GluA1 (pS845-GluA1), total GluA1 and GAPDH (bottom panel; loading control). ***E***, Quantification in pS845-GluA1 and GluA1 levels in response to AKAP150 knock-down compared with basal (*n* = 3, *p = 0.0057 vs basal; #*p* = 0.0070 vs basal). ***F***, AKAP150 overexpression does not modify S845-GluA1 phosphorylation and total GluA1 protein levels. Representative blot images showing AKAP150 (top panel; as a control of overexpression), phosphorylated GluA1 (pS845-GluA1), total GluA1 and GAPDH (bottom panel; loading control) levels. ***G***, Quantification of pS845-GluA1 and GluA1 in response to AKAP150 overexpression compared with basal (*n* > 3). ***H***, Representative confocal images of dendrites show GluA1 (magenta), presynaptic VGlut1 (gray), and postsynaptic PSD95 (blue) levels after knocking down AKAP150 compared with basal condition (Ø; empty vector). GFP expression (green) is used as a control of transduction. Down-regulation of endogenous AKAP150 reduced dendritic and synaptic GluA1 intensity. Bottom panels in ***H*** show the merge for GFP/GluA1/PSD95/VGlut1. ***I***, Quantification of GluA1 (GluA1) intensity normalized to basal. AKAP150 knockdown expression reduced dendritic (o) and synaptic (ν) GluA1 (*n* > 30 dendrites of different neurons from five independent cultures, *p < 0.0001 vs basal; #*p* < 0.0001 vs basal). ***J***, Surface GluA1 levels on cLTD in presence of shAKAP150. Representative blot images showing decreased levels of surface GluA1 after cLTD in presence of shAKAP150 (sGluA1; ∼110-kDa band, top panel) related to total GluA1 (tGluA1; ∼110-kDa band, middle panel). GAPDH was used as a loading control (∼36-kDa band, bottom panel). ***K***, Quantification of surface GluA1 changes in response to cLTD in presence of shAKAP150 compared with basal condition (*n* = 3, *p = 0.0030 for cLTD, *p = 0.0012 for shAKAP150+cLTD, *p = 0.0018 for empty+cLTD and *p = 0.0141 for shAKAP150 vs basal). ***L***, Quantification of AKAP150 in response to cLTD in presence of shAKAP150 compared with basal condition (*n* = 3, *p =* *0.0080 for cLTD, *p <* *0.0001 for shAKAP150+cLTD, *p =* *0.0080 for empty+cLTD and *p =* *0.0003 for shAKAP150 vs basal). Scale bars = 5 μm. Bars represent mean ± SEM.

**Figure 7.**
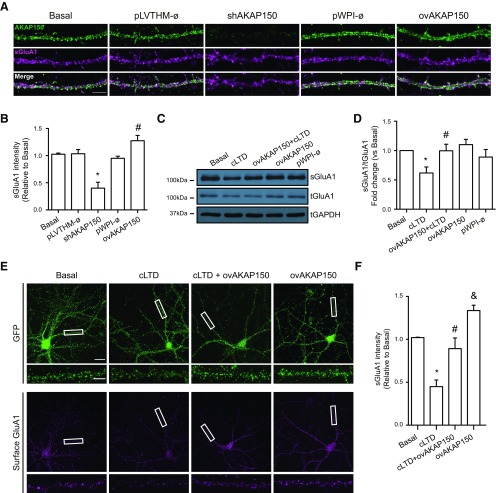
Overexpression of AKAP150 blocked cLTD-mediated GluA1-AMPARs endocytosis. ***A***, Representative confocal images of dendrites showing AKAP150 silencing (shAKAP150) and overexpressing (ovAKAP150) effect on surface GluA1 expression. AKAP150 (green) and surface GluA1 (magenta). Bottom panels, Merge for AKAP150/sGluA1. Knocking down endogenous AKAP150 reduced and AKAP150 overexpression increased surface GluA1 intensity. Empty lentiviral vector (Ø; pLVTHM or pWPI) was used as a control. Scale bar = 5 μm. ***B***, Quantification of surface GluA1 intensity (sGluA1) after silencing or overexpressing AKAP150 (*n* > 12 dendrites of different neurons from four independent cultures, *p <* *0.0001, #*p *=* *0.0419 vs basal). ***C***, cLTD was induced in primary neurons overexpressing AKAP150 and surface proteins were biotinylated and pulled-down. Representative blot images show surface GluA1 (top panel; sGluA1), total GluA1 (middle panel; tGluA1) and GAPDH levels (bottom panel; loading control). ***D***, Quantification of surface GluA1 intensity (sGluA1) in response to cLTD without or with overexpression of AKAP150 compared with basal (*n* = 6, *p =* *0.0173 vs basal; #*p *=* *0.0185 vs cLTD). ***E***, Representative confocal images show surface GluA1 and GFP staining on primary neurons overexpressing AKAP150 after cLTD induction. Separated panels from the boxed regions have been magnified for dendrite clarity (bottom panels). AKAP150 overexpression prevents cLTD-dependent surface GluA1 reduction compared with control cells expressing GFP. ***F***, Quantification of surface GluA1 intensity (sGluA1) in response to cLTD without or with overexpression of AKAP150 (*n* = 10 dendrites from 10 independent cultures, *p <* *0.0001 vs basal; #*p *=* *0.0016 vs cLTD; &*p *=* *0.0285 vs basal). Scale bars = 20 μm (top panels) and 5 μm (bottom panels). Bars represent mean ± SEM.

Synaptic distribution of AKAP150 is a key factor for synaptic expression of AMPARs (Tavalin et al., 2002). Previous reports have shown that removal of AKAP150 from synapses, toward the cytosol in dendritic shafts or soma, elicits synaptic AMPARs endocytosis (Bhattacharyya et al., 2009; Keith et al., 2012). In this context, we explored AKAP150 silencing effect on AMPAR. Using shRNA-mediated acute knock-down of endogenous AKAP150 (shAKAP150), we were able to effectively reduce AKAP150 expression in cultured neurons (Fig. 6*A*). Interestingly, whereas cLTD produces the degradation of AKAP150 (Fig. 1*F*,*G*), PSD95 (Fig. 3*C*,*D*), and SAP97 (data not shown), knock-down of endogenous AKAP150 did not induce significant changes in PSD95 or SAP97 levels (Fig. 6*B*,*C*; shAKAP150: 1.070 ± 0.112 and 0.873 ± 0.145, respectively). However, AKAP150 silencing decreased S845-GluA1 phosphorylation and GluA1 total levels (Fig. 6*D*,*E*; shAKAP150: 0.327 ± 0.101; *p =* *0.0057 and 0.516 ± 0.073; #*p *=* *0.0070, respectively, vs Ø). Consistent with the results observed by immunoblotting, AKAP150 knock-down reduced both synaptic and dendritic GluA1 levels (Fig. 6*H*,*I*; shAKAP150: 0.432 ± 0.088; *p <* *0.0001 and 0.445 ± 0.023; #*p *<* *0.0001, respectively), followed by a decrease in surface GluA1 expression (Fig. 7*A*,*B*; shAKAP150: 0.401 ± 0.109; *p <* *0.0001 vs basal). Next, we wanted to test whether this reduction in surface GluA1 by AKAP150 silencing could occlude further AMPAR removal after cLTD induction. We induced cLTD in AKAP150-silenced neurons and surface GluA1 levels were analyzed. We observed that after cLTD in shAKAP150 conditions, further reduction of GluA1 surface levels was observed (Fig. 6*J*,*K*; shAKAP150: 0.472 ± 0.115; *p =* *0.0141, cLTD: 0.287 ± 0.081; *p =* *0.0030 and shAKAP150 + cLTD: 0.152 ± 0.053; *p =* *0.0012 vs basal). These results clearly show that silencing endogenous AKAP150 decreases Ser-845 phosphorylation, GluA1 levels and consequently reduces GluA1-containing AMPARs recruitment to synapses and surface expression. Remaining levels of AKAP150, under shAKAP150 conditions, seem to be enough to partially keep anchored AMPAR to the membrane and, after cLTD, its degradation still contribute to increase GuA1 internalization (Fig. 6*J*,*L*; shAKAP150: 0.279 ± 0.018; *p =**0.0003, cLTD: 0.517 ± 0.070; *p =* *0.0080 and shAKAP150 + cLTD: 0.131 ± 0.027; *p <* *0.0001 vs basal).

### Overexpression of AKAP150 blocks cLTD-mediated AMPARs endocytosis

Above results show that a decrease in AKAP150 endogenous levels either by cLTD induction, shRNA-mediated knock-down or both, triggers GluA1-containing AMPARs endocytosis. Therefore, we studied whether overexpression of AKAP150 was able to rescue AMPARs endocytosis produced after cLTD. According to previous studies showing that AKAP150 overexpression somehow increases basal AMPAR surface expression (Bhattacharyya et al., 2009), neurons overexpressing AKAP150 showed a slight no significant increase in Ser-845 phosphorylation without affecting GluA1 total levels (Fig. 6*F*,*G*; ovAKAP150: 1.180 ± 0.047 and 0.963 ± 0.125, respectively). However, AKAP150 overexpression did elevate surface GluA1 labeling (∼24%) compared with basal (Fig. 7*A*,*B*; ovAKAP150: 1.274 ± 0.098; #*p* = 0.0419). When cLTD was induced in AKAP150 overexpressing neurons, surface GluA1 levels remained unchanged (Fig. 7*C*,*D* and *E*,*F*; ovAKAP150+ cLTD: 0.996 ± 0.112; #*p *=* *0.0185 vs cLTD and ovAKAP150+ cLTD: 0.893 ± 0.125; #*p *=* *0.0016 vs cLTD).

## Discussion

It is well known that modulation of AMPAR function and membrane trafficking, in and out the synapses, is a major contributor to synaptic plasticity processes, such as LTP or LTD, that are believed to underlie learning and memory (Huganir and Nicoll, 2013). Whereas LTP has been associated to the insertion of AMPARs in the postsynaptic membrane, the synaptic AMPARs endocytosis drives NMDA-dependent LTD (Lledo et al., 1998; Carroll et al., 1999; Takumi et al., 1999; Shi et al., 2001). It has been previously described that the phosphorylated state of AMPARs modulates their insertion or removal from synaptic membranes (Lee et al., 1998, 2000, 2003, 2010; Chung et al., 2000; Esteban et al., 2003; Boehm et al., 2006; Oh et al., 2006; Delgado et al., 2007; Lu et al., 2010; Coultrap et al., 2014). Among AMPARs subunits, it is believed that GluA1 trafficking in and out the synapse depends on neuronal activity, while GluA2 subunit seems to be constitutively regulated in a neuronal activity-independent manner (Passafaro et al., 2001; Shi et al., 2001). Phosphorylation status of GluA1 on Ser-845 seems to determine synaptic localization of GluA1 AMPARs during LTP or LTD processes. In this way, PKA-mediated Ser-845 phosphorylation facilitates AMPARs trafficking to the synapses favoring LTP (Oh et al., 2006; Makino et al., 2011) whereas Ser-845 dephosphorylation by CaN has been shown to promote AMPARs endocytosis associated to hippocampal LTD (Mulkey et al., 1994; Beattie et al., 2000; Lee et al., 2000).

Several studies have reported that the scaffolding protein AKAP79/150 has a prominent role in the reciprocal modulation of PKA and CaN activities at the synapse and therefore in the regulation of AMPARs cycling in and out the synapses during LTP and LTD (Tavalin et al., 2002; Lu et al., 2008; Jurado et al., 2010). AKAP79/150 forms a complex with PKA and CaN (Bauman et al., 2004; Jurado et al., 2010) that binds the MAGUK scaffold proteins PSD95 and SAP97, linking the AKAP79/150 complex to AMPARs (Colledge et al., 2000; Bhattacharyya et al., 2009; Robertson et al., 2009). Thus, a disruption in the interaction between these proteins in the PSD could lead to an alteration in plasticity of glutamatergic synapses. For instance, PSD95 knock-down by shRNA or inhibition of PSD95 binding to AKAP150, blocks NMDA-dependent LTD endocytosis of synaptic AMPARs in cultured neurons (Bhattacharyya et al., 2009). In the present work, we studied the eventual changes in AKAP150 levels caused by cLTD and whether these changes are involved in synaptic AMPARs endocytosis triggered by cLTD in neuronal cultures.

**Figure 8.**
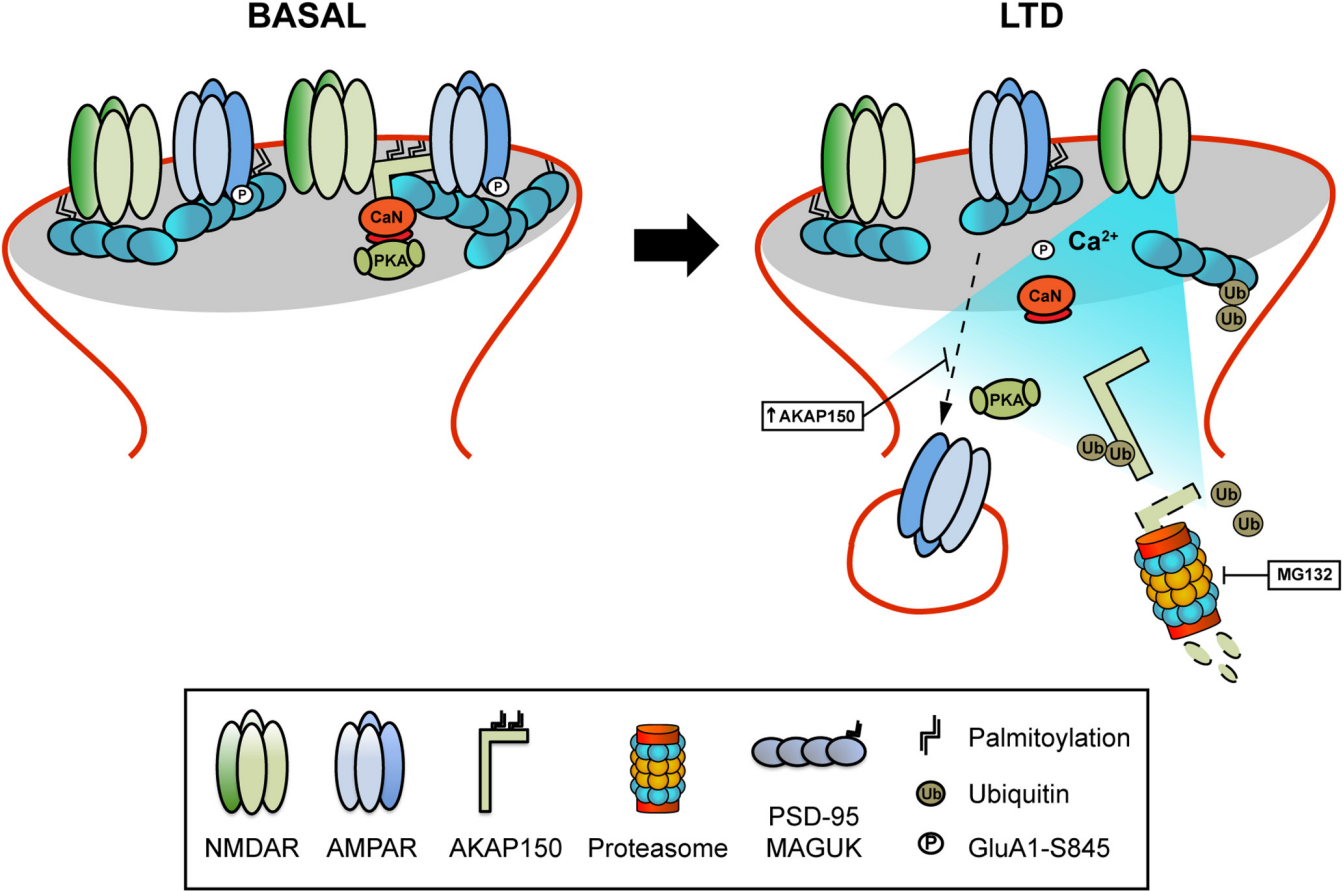
Model for LTD-mediated AKAP150 degradation through the proteasome system. Under basal conditions, a pool of palmitoylated AKAP150 is anchoring and regulating PKA and CaN function in dendritic spines. After LTD induction, a weak and extended Ca^2+^ influx depalmitoylate and remove AKAP150 from spines. This removal promotes CaN-mediated dephosphorylation and consequent endocytosis of GluA1-AMPARs. During this process AKAP150 is ubiquitinated and degraded via proteasome. The presence of 20S proteasome inhibitor MG132 blocks not only LTD-mediated AKAP150 degradation but also its removal from spines. Maintenance of AKAP150 levels is sufficient to prevent LTD-induced GluA1-AMPARs endocytosis suggesting that AKAP150 degradation by the proteasome could be an important factor in the regulation of surface GluA1-AMPARs at spines.

Previous results suggested that during LTD, AKAP79/150, together with PKA, translocate away from PSD95 and synaptic spines (Gomez et al., 2002; Smith et al., 2006). This translocation is believed to favor CaN activity promoting the dephosphorylation of postsynaptic substrates, such as GluA1 and leading to synaptic AMPARs endocytosis (Smith et al., 2006). Our results show that besides AKAP79/150 redistribution away from the synapses, cLTD produces an additional and significant proteasome-dependent decrease in total AKAP150 levels that it is accompanied by a reduction in surface and total GluA1 levels. Furthermore, our data also show that the addition of shAKAP150 does not blocks NMDA-dependent LTD endocytosis of synaptic AMPAR, indicating a differential role of AKAP150, PSD95, and its interaction on AMPAR endocytosis in LTD (Bhattacharyya et al., 2009). Moreover, the maintenance of AKAP150 levels by overexpression is sufficient to prevent GluA1-AMPARs endocytosis associated to cLTD in cultured neurons. A causative relationship between AKAP150 levels decrease and AMPARs endocytosis is also supported by our data using shAKAP150 together with AKAP150 overexpression; whereas AKAP150 silencing produced the expected dephosphorylation of GluA1 Ser-845 and the endocytosis of GluA1-AMPARs, overexpression of AKAP150 increased GluA1 Ser-845 phosphorylation and surface GluA1 levels. Precisely, overexpression of AKAP150 not only drives more AMPAR to the membrane but can also block cLTD-mediated GluA1-AMPARs endocytosis, indicating that the presence of AKAP150 is increasing retention/stabilization at synaptic locations. Further work remains to be done to address whether changes in AKAP150 could, and how, also affect other scaffolding proteins. Moreover, since LTD triggers AKAP150 degradation, AKAP150 plays an important role regulating LTP processes (Sanderson and Dell'Acqua, 2011) and LTP is associated with up-regulation of AKAP150 gene expression (Génin et al., 2003), we postulate that AKAP150 degradation would impair subsequent LTP. This is supported by our data showing that cLTD prevent cLTP induction due to a failure in normal AKAP150 levels recovery. Surprisingly, prior cLTP induction as a strategy to retain AKAP150 levels and block cLTD effect on GluA1-AMPAR was unproductive according to our data. It is possible that cLTP induction 1 h before cLTD is not enough to protect AKAP150 from cLTD. However, from our images it seems that there may be more AKAP150 concentrated at the plasma membrane after cLTP induction, with bigger and more intense clusters than in basal conditions in agreement with the notion that LTP recruits more AKAP150 to the plasma membrane (Purkey et al., 2018). Additional experiments are needed to explore this possibility at different time points. Based on these data, we propose that cLTD has a double effect on AKAP79/150 that is responsible for AMPARs dephosphorylation and endocytosis: a translocation of AKAP79/150 out of the synapses that precedes its synaptic or extra-synaptic degradation by proteasome (Fig. 8). Since, cLTD would prevent cLTP induction depending on AKAP150 levels, it is feasible to hypothesize that the intensity of cLTD stimulus could determine the extent of AKAP150 degradation and the recovery time necessary for cLTP induction, although additional experiments are needed.

Our results indicating that AKAP150 levels influence surface expression and phosphorylation of AMPARs, support previous reports showing that the expression levels of other components of the PSD complex regulate synaptic distribution and function of AMPARs. For instance, it has been described that overexpression of PSD95 in cultured neurons increases synaptic AMPARs delivery (El-Husseini et al., 2000). By contrast, shRNA-mediated knock-down of PSD95 decreases synaptic AMPARs and enhances LTD (Elias et al., 2006; Schlüter et al., 2006; Ehrlich et al., 2007; Bhattacharyya et al., 2009). Similar results have been observed with SAP97 (Rumbaugh et al., 2003; Schlüter et al., 2006; Howard et al., 2010). Interestingly, we have also observed that PSD95 and SAP97 (unpublished data) levels decrease after cLTD induction. However, shRNA-mediated silencing of endogenous AKAP150 did not change PSD95 and SAP97 levels in cultured neurons, suggesting that although cLTD triggers a coordinated down-regulation of these proteins, an indirect cross-regulation mechanism may occur between them.

It has been reported a key role of CaN in the modulation of AMPARs trafficking in and out the synapses mediated by its capacity to dephosphorylate AMPARs and other proteins in the PSD (Smith et al., 2006; Jurado et al., 2010). Here, we have reported that CaN activation is not necessary for cLTD-mediated AKAP150 degradation by proteasome. Therefore, CaN would be able to favor cLTD by enhancing synaptic AMPARs endocytosis but it would not participate in the induction of AKAP79/150 degradation. In this context, It has been described that activity-dependent palmitoylation of AKAP79/150 is necessary for synaptic targeting of AKAP79/150 and cLTP induction (Keith et al., 2012). In addition, cLTD activates CaMKII regulating depalmitoylation and synaptic removal of AKAP79/150 (Woolfrey et al., 2018) being feasibly a prerequisite for posterior degradation in the proteasome.

At present, the mechanism through which cLTD induces proteasome-dependent AKAP150 degradation is unknown. However, It is well known that AKAP150 is highly ubiquitinated and can be regulated by ubiquitin-proteasome system depending on synaptic activity (Ehlers 2003; Jia et al., 2008). Moreover, it has recently been described that Nedd4 ubiquitinates (Schwarz et al., 2010; Lin et al., 2011) and USP46 deubiquitinates (Huo et al., 2015) AMPARs. Some controversy exists about the role of cLTD on ubiquitination-proteasome system and synaptic function. Previous data from Citri and colleagues showed that cLTD-mediated AMPAR endocytosis requires ubiquitination but not proteasomal degradation (Citri et al., 2009), while our results suggest that both ubiquitin and proteasome processes are involved in cLTD-mediated AKAP150 degradation. AKAP150 seems to be part of a complex of synaptic proteins regulated by proteasome system in the molecular mechanisms of LTD. Here, we also find involved PSD95 and GluA1 (data not shown) in proteasomal-mediated degradation indicating that it could be a more general effect on synaptic proteins. Furthermore, restoration of AKAP150 levels by overexpression was able to rescue cLTD-dependent GluA1-AMPAR endocytosis. This finding highlights the importance of AKAP150 levels in excitatory synapses along with their interaction to other scaffolding proteins through the MAGUK binding domain (Robertson et al., 2009). Nevertheless, further work should be addressed interfering selectively with AKAP150 cLTD-induced ubiquitination and to prevent aspects of LTD expression or whether Nedd4 and/or USP46 proteins also regulate AKAP79/150 ubiquitination and their activities under cLTD condition to further support an AKAP150-degradation centric model in control of AMPAR endocytosis in LTD.

In summary, consistent with previous results describing AKAP79/150 translocation away from synapses in cLTD (Gomez et al., 2002; Smith et al., 2006; Woolfrey et al., 2018), our data show that proteasome-dependent AKAP150 degradation in cLTD could be an important mechanism in the regulation of surface GluA1-AMPARs at excitatory synapses (Fig. 8).

10.1523/ENEURO.0218-19.2020.supplement Statistical Table Supplementary Statistical Table. Download Statistical Table, DOCX file.

## Synthesis

Reviewing Editor: Pablo Castillo, Albert Einstein College of Medicine Decisions are customarily a result of the Reviewing Editor and the peer reviewers coming together and discussing their recommendations until a consensus is reached. When revisions are invited, a fact-based synthesis statement explaining their decision and outlining what is needed to prepare a revision will be listed below. The following reviewer(s) agreed to reveal their identity: NONE.

From the online discussion the reviewers agree the revised manuscript has clearly improved, but a number of concerns remain, including the quality of the culture neurons with images showing high heterogeneity (i.e. abundant PSD95-positive neurons but no AKAP150 expression). They understand the finding that AKAP150 is degraded by the proteasome following LTD is a novel finding. However, the reviewers' main concern is that your findings can only establish correlations and as a result the precise role of AKAP150 in LTD remains unclear. You will have to directly address this issue for your manuscript to have a chance in eNeuro. Alternatively, you may want to significantly tone down your main conclusion that proteasomal-mediated degradation of AKAP150 is required for AMPAR endocytosis during cLTD, as bluntly put in the title of your manuscript. Below, please find the reviewers' specific concerns, which I strongly recommend you should address in full.

---------------------------------------------------------------------------

REVIEWER 1

I commend the authors for their efforts as the present version is a much improved manuscript. Authors have addressed satisfactorily all my concerns with new and relevant experiments that contribute to a better understanding of the role of AKAP150 in regulating synaptic plasticity.

I have a few minor comments that need to be solved prior publication:

Figs 1A and 5A: There is a typo in the NMDA treatment, it should say 50 microM instead of 50 mM. Also calcium chelators are no indicated in the scheme in Fig 1A. Please correct concentrations in the scheme.

Fig 2C: The magnification used in the cut-out panel for highlighting the dendrite in baseline conditions seems higher than the corresponding one for cLTD. Please check and use images with the same magnification settings. Also these pictures clearly show that some neurons express high levels of PSD95 but not AKAP150, suggesting some heterogeneity of AKAP150 expression in cultured neurons. Also from this image is difficult to appreciate a reduction of AKAP150 or PSD-95 upon cLTD. However, this effect is more clear in Fig 3C. These results suggest the efficacy of the cLTD protocol varies depending on the cultures. A comment indicating these issues will be a nice addition to the Methods section.

Fig 5: As mentioned by the authors, it is interesting that inducing cLTP prior cLTD does not prevent AKAP150 degradation, however it may not be as surprising as potentiated synapses are still capable of expressing LTD as shown in de-potentiation experiments elicited by electrical stimulation. Although it may not be significant, from the images it seems that there may be more AKAP150 concentrated at the plasma membrane after cLTP induction, with bigger and more intense clusters than in control conditions in agreement with the notion that LTP recruits more AKAP150 to the plasma membrane.

REVIEWER 2

Manuscript eN-NWR-0218-19R1 has been revised to include additional data that clarifies questions relating to the time course and the specificity of AKAP150 degradation following cLTD. Despite these and other improvements, major concerns remain regarding experimental approach and data interpretation which still preclude a recommendation for publication in its present form. These concerns are detailed below:

The authors have clarified the age and prepration methods for the cultured hippocampal neurons featured in the manuscript. Though cell age is more in line with expectations for mature neurons, the appearance of the representative images still shows a lack of dense, mature spine structures. Even though the linear density of PSD-95 puncta may appear normal, from the images provided, the majority of these synapses seem to be located on the dendrite shaft and not in spines, which seem to be largely absent. Accordingly, the authors should include cells expressing a fluorescent protein cell fill, allowing for visualization and quantification dendritic spine morphology and linear density. These data would allay fears regarding the cellular identity and maturity of the neurons quantified.

In particular, it is troublesome that there are many dendrites from apparently PSD-95-positive, but AKAP150-negative cells depicted in the background of Figs 2C and 3E. Most cells on the dish should be AKAP150+. Occasionally, large interneurons with limited secondary and tertiary dendritic branching and few spines can exhibit strong AKAP150 expression, which may result in staining so bright that it obscures signal in neighboring cells. Finding fields of neurons that don't feature these bright cells will likely reveal AKAP150 staining in all pyramidal cells; these cells may exhibit more normal spine morphology and number. As its stands now, the AKAP150 expressing neurons the authors have focused their analyses on may actually be inhibitory interneurons and not excitatory pyramidal cells.

Fig 6H top middle and right panels purportedly show a GFP cell fill, but it looks a lot like the more punctate appearance of AKAP150 staining/overexpression as opposed to the more uniform distribution of GFP expected for a cell fill. Is this labeled correctly? And if so, what is the explanation for its punctate appearance?

Fig 7E is supposed to show surface GluA1, yet there is a great deal of signal in the cell body outside the nucleus which is where intracellular AMPARs in the ER would be prominently localized. Are there controls for this surface labeling under permeabilized vs. non-permeabilized conditions that could be provided to increase confidence that the panels indeed show surface labeling and not total labeling?

Fig 4C, D are very difficult to interpret. I understand that Ub blotting is challenging, but I cannot understand how the quantification presented is derived from the supplied blots. Also, if there is more Ub-AKAP150 after cLTD, wouldn't that suggest that AKAP is more resistant to degradation than other ubiquitinated proteins because cLTD actually decreased the signal for total ubiquitinated proteins?

Finally, and perhaps most importantly, it is not clear that AKAP150 degradation following cLTD provides additional function above and beyond its trafficking away from the synapse. The default hypothesis would be that AKAP overexpression would provide additional enrichment in many domains, including the synapse, the extrasynaptic plasma membrane, and endosomes, where it could play its role in GluA1 phosphorylation and maintenance on the cell surface via structural interactions and/or PKA signaling. At minimum, data on the effect of cLTD on overexpressed AKAP150 levels needs to be shown and discussed i.e. does this AKAP150 overexpression actually block cLTD-induced AKAP degradation and/or translocation from the plasma membrane? Further, since many other synaptic components are degraded on roughly the same time scale as AKAP150 (GluA1 itself and PSD95), it is difficult to identify degradation AKAP as uniquely important for cLTD. These limitations and alternate possibilities should be acknowledged and discussed. Though no doubt challenging, the authors would need to selectively interfere with AKAP cLTD-induced ubiquitination and prevent aspects of LTD expression in order to make a stronger case for an AKAP-degradation centric model in control of AMPAR endocytosis in LTD.

The effects on chronic viral shRNA knockdown of AKAP150 expression leading to reduced basal GluA1 levels could also be quite indirect, influenced by homeostatic mechanisms, and not directly related to LTD mechanisms as proposed. These findings showing such a strong reduction in basal GluA1 levels are also surprising given that multiple previous studies using AKAP150 RNAi knockdown or KO in cultures and slices found no decreases in basal AMPAR transmission (increases were actually observed in some cases), little of no decreases in basal S845 phosphorylation, and little or no change in basal AMPAR levels or surface expression (see Tunquist et al., 2008 PNAS, Bhattacharyya et al. 2009 Nat Neurosci, Jurado et al. Nat Neurosci 2010, Weisenhaus et al., 2010 PlosOne; Keith et al., 2012 J Neurosci, Zhang...Hell et al. 2013 J Biol Chem, Sanderson et al., 2018 J Neurosci). Also, in several of these studies exogenous expression/overexpression of either AKAP150 or AKAP79 was used and found not to interfere with AMPAR endocytosis and/or functional LTD (see Bhattacharyya et al. 2009 Nat Neurosci, Jurado et al. Nat Neurosci 2010, Keith et al., 2012 J Neurosci). These inconsistencies with the literature need to be addressed.

Thus, at this point, while the authors provide strong correlative data very nicely showing that AKAP150 degradation occurs at later time points following cLTD, they fail to provide convincing causative data showing that this AKAP150 degradation is actually directly required for any aspect of cLTD (i.e. GluA1 surface decreases, S845 dephosphorylation, actual depression of AMPAR EPSCs, PSD-95 degradation, spine shrinkage etc.). Thus, without additional causative data, such as showing that cLTD is prevented by mutating sites of ubiquitination in AKAP150 that mediate its degradation, the overall conclusion of the study would need to softened considerably and the title of the paper would need to be changed to "Proteasomal-mediated degradation of AKAP150 accompanies AMPAR endocytosis during cLTD".
