# Supplementary material for: Proteasomal-Mediated Degradation of AKAP150 Accompanies AMPAR Endocytosis during cLTD
Source: eNeuro. 2020 Mar 31;7(2):ENEURO.0218-19.2020. doi: 10.1523/ENEURO.0218-19.2020 (PMC7163082; doi:10.1523/ENEURO.0218-19.2020)
Supplement: Statistical Table — Supplementary Statistical Table. Download Statistical Table, DOCX file. [file enu-eN-NWR-0218-19-s02.docx]

**Statistical Table**

| **Figure** | **Graph** | **Data structure** | **Type of test** | **p-values** | **Power (95% C.I. of diff)** |
| --- | --- | --- | --- | --- | --- |
| Figure 1 | 1C, ◼, * | Normal distribution | One-way ANOVA; Bonferroni’s *post hoc* test | < 0.0001 | 0,2539 to 0,8473  0,4204 to 1,014  0,4969 to 0,7734  0,4631 to 0,8669 |
| Figure 1 | 1C, 🞏, * | Normal distribution | One-way ANOVA; Bonferroni’s *post hoc* test | 0,0189  < 0.0001 | 0,02597 to 0,3921  0,2902 to 0,5217  0,4020 to 0,7681 |
| Figure 1 | 1E, * | Normal distribution | One-way ANOVA; Bonferroni’s *post hoc* test | 0,0310  < 0.0001 | 0,02005 to 0,5283  0,2774 to 0,7657  0,4726 to 1,205 |
| Figure 1 | 1G, * | Normal distribution | One-way ANOVA; Bonferroni’s *post hoc* test | 0,0021  < 0.0001 | 0,07150 to 0,4002  0,5203 to 0,7282  0,5586 to 1,046 |
| Figure 1 | 1I, * | Normal distribution | One-way ANOVA; Bonferroni’s *post hoc* test | 0.0187 | 0,09080 to 0,8595 |
| Figure 1 | 1I, # | Normal distribution | One-way ANOVA; Bonferroni’s *post hoc* test | 0.0377 | -0,7942 to -0,02557 |
| Figure 1 | 1K, * | Normal distribution | One-way ANOVA; Bonferroni’s *post hoc* test | < 0.0001 | 0,4233 to 0,7744 (cLTD)  0,4450 to 0,7960 (cLTD+FK506) |
| Figure 2 | 2B, * | Normal distribution | One-way ANOVA; Bonferroni’s *post hoc* test | 0.0036 | 0,1950 to 0,8749 |
| Figure 2 | 2B, # | Normal distribution | One-way ANOVA; Bonferroni’s *post hoc* test | 0.0395 | -0,6971 to -0,01725 |
| Figure 2 | 2D, * | Normal distribution | One-way ANOVA; Bonferroni’s *post hoc* test | 0,0083 | 0,1120 to 0,8802 |
| Figure 2 | 2D, # | Normal distribution | One-way ANOVA; Bonferroni’s *post hoc* test | < 0.0001 | -1,577 to -0,9790 |
| Figure 2 | 2E, * | Normal distribution | One-way ANOVA; Bonferroni’s *post hoc* test | 0.0150 | 0,05031 to 0,6173 |
| Figure 2 | 2E, # | Normal distribution | One-way ANOVA; Bonferroni’s *post hoc* test | < 0.0001 | -1,009 to -0,5287 |
| Figure 3 | 3B,* | Normal distribution | One-way ANOVA; Bonferroni’s *post hoc* test | 0,0125 | 0,1070 to 0,8214 |
| Figure 3 | 3B,# | Normal distribution | One-way ANOVA; Bonferroni’s *post hoc* test | 0,0014 | -1,078 to -0,3064 |
| Figure 3 | 3D,* | Normal distribution | One-way ANOVA; Bonferroni’s *post hoc* test | 0,0092 | 0,1605 to 0,9347 |
| Figure 3 | 3D,# | Normal distribution | One-way ANOVA; Bonferroni’s *post hoc* test | 0,0091 | -0,9357 to -0,1615 |
| Figure 3 | 3F,* | Normal distribution | One-way ANOVA; Bonferroni’s *post hoc* test | 0,0083 | 0,1120 to 0,8802 |
| Figure 3 | 3F,# | Normal distribution | One-way ANOVA; Bonferroni’s *post hoc* test | <0.0001 | -0,9668 to -0,3597 |
| Figure 3 | 3G,* | Normal distribution | One-way ANOVA; Bonferroni’s *post hoc* test | 0.0150 | 0,05031 to 0,6173 |
| Figure 3 | 3G,# | Normal distribution | One-way ANOVA; Bonferroni’s *post hoc* test | <0.0001 | -0,8059 to -0,3399 |
| Figure 3 | 3H,* | Normal distribution | One-way ANOVA; Bonferroni’s *post hoc* test | 0,0005 | 6380 to 11536 |
| Figure 3 | 3H,# | Normal distribution | One-way ANOVA; Bonferroni’s *post hoc* test | 0,0002 | -13851 to -8694 |
| Figure 4 | 4B, * | Normal distribution | One-way ANOVA; Bonferroni’s *post hoc* test | 0.0355 | 0,02099 to 0,6478 |
| Figure 4 | 4B, # | Normal distribution | One-way ANOVA; Bonferroni’s *post hoc* test | 0.0183 | -0,6873 to -0,06048 |
| Figure 4 | 4D, * | Normal distribution | One-way ANOVA; Bonferroni’s *post hoc* test | 0.0026 | -20,41 to -5,591 |
| Figure 5 | 5C, * | Normal distribution | One-way ANOVA; Bonferroni’s *post hoc* test | < 0.0001  0.0009 | 0,4227 to 0,7415  -0,5065 to -0,1431 |
| Figure 5 | 5E,* | Normal distribution | One-way ANOVA; Bonferroni’s *post hoc* test | < 0.0001 | 0,3047 to 0,6061  0,4446 to 0,7553  0,5049 to 1,005  0,4725 to 0,8291 |
| Figure 5 | 5G, * | Normal distribution | One-way ANOVA; Bonferroni’s *post hoc* test | 0.0004 | 0,2308 to 0,8815 |
| Figure 5 | 5G, # | Normal distribution | One-way ANOVA; Bonferroni’s *post hoc* test | 0.0017 | 0,1818 to 0,8847 |
| Figure 6 | 6E, ◼, * | Normal distribution | Unpaired Student’s *t* test (Two-tailed P value) | 0.0057 | -1,020 to -0,3260 |
| Figure 6 | 6E, 🞏, # | Normal distribution | Unpaired Student’s *t* test (Two-tailed P value) | 0.0070 | -0,7473 to -0,2204 |
| Figure 6 | 6I, ◼, * | Normal distribution | One-way ANOVA; Bonferroni’s *post hoc* test | < 0.0001 | 0.3356 to 0.8010 |
| Figure 6 | 6I, 🞏, # | Normal distribution | One-way ANOVA; Bonferroni’s *post hoc* test | < 0.0001 | 0,3946 to 0,7147 |
| Figure 6 | 6K,* | Normal distribution | One-way ANOVA; Bonferroni’s *post hoc* test | 0,0030  0,0012  0,0141  0,0018 | 0,3108 to 1,115  0,4461 to 1,251  0,1255 to 0,9300  0,3791 to 1,184 |
| Figure 6 | 6L,* | Normal distribution | One-way ANOVA; Bonferroni’s *post hoc* test | 0,0080  < 0,0001  0,0080  0,0003 | 0,1192 to 0,8470  0,5051 to 1,233  0,1192 to 0,8470  0,3568 to 1,085 |
| Figure 7 | 7B, * | Normal distribution | One-way ANOVA; Bonferroni’s *post hoc* test | < 0.0001 | 0,4157 to 0,8340 |
| Figure 7 | 7B, # | Normal distribution | One-way ANOVA; Bonferroni’s *post hoc* test | 0.0419 | -0,4893 to -0,008035 |
| Figure 7 | 7D, * | Normal distribution | One-way ANOVA; Bonferroni’s *post hoc* test | 0.0173 | 0,05882 to 0,7062 |
| Figure 7 | 7D, # | Normal distribution | One-way ANOVA; Bonferroni’s *post hoc* test | 0.0185 | -0,7024 to -0,05508 |
| Figure 7 | 7F, * | Normal distribution | One-way ANOVA; Bonferroni’s *post hoc* test | < 0.0001 | 0,2727 to 0,8687 |
| Figure 7 | 7F, # | Normal distribution | One-way ANOVA; Bonferroni’s *post hoc* test | 0.0016 | -0,7336 to -0,1518 |
| Figure 7 | 7F, & | Normal distribution | One-way ANOVA; Bonferroni’s *post hoc* test | 0.0285 | -0,6047 to -0,02644 |
